# LAD-RCNN: A Powerful Tool for Livestock Face Detection and Normalization

**DOI:** 10.3390/ani13091446

**Published:** 2023-04-24

**Authors:** Ling Sun, Guiqiong Liu, Huiguo Yang, Xunping Jiang, Junrui Liu, Xu Wang, Han Yang, Shiping Yang

**Affiliations:** 1 Key Laboratory of Smart Farming for Agricultural Animals, Wuhan 430070, China; sunling@webmail.hzau.edu.cn; 2 Laboratory of Small Ruminant Genetics, Breeding and Reproduction, College of Animal Science and Technology, Huazhong Agricultural University, Wuhan 430070, China; liuguiqiong@mail.hzau.edu.cn (G.L.); liujunrui@webmail.hzau.edu.cn (J.L.); wangxu513@webmail.hzau.edu.cn (X.W.); yanghan587@webmail.hzau.edu.cn (H.Y.); yangshiping@webmail.hzau.edu.cn (S.Y.); 3 Key Laboratory of Agricultural Animal Genetics, Breeding and Reproduction of the Ministry of Education, Wuhan 430070, China; 4 Institute of Animal Husbandry, Xinjiang Academy of Animal Sciences, Urumqi 830013, China; yhg760924@163.com

**Keywords:** livestock face detection, rotation angle detection, livestock face normalization, face recognition

## Abstract

**Simple Summary:**

Livestock face recognition has become one of the research hotspots; animal face recognition refers to identification recognition based on livestock face images. Face normalization is an important step in face recognition, which refers to extracting animal facial images from raw images and aligning them through methods such as rotation. However, it appears that no previous studies have focused on livestock face normalization. To address this gap in research, a new approach has been developed called the lightweight angle detection and region-based convolutional network (LAD-RCNN). LAD-RCNN is designed to detect livestock faces and their rotation angles with arbitrary directions in one stage. With the use of LAD-RCNN, livestock face normalization can be easily achieved through techniques such as clipping, rotation, and scaling based on the detected face location and rotation angle. Overall, LAD-RCNN offers promise for improving the accuracy and efficiency of livestock face recognition.

**Abstract:**

With the demand for standardized large-scale livestock farming and the development of artificial intelligence technology, a lot of research in the area of animal face detection and face identification was conducted. However, there are no specialized studies on livestock face normalization, which may significantly reduce the performance of face identification. The keypoint detection technology, which has been widely applied in human face normalization, is not suitable for animal face normalization due to the arbitrary directions of animal face images captured from uncooperative animals. It is necessary to develop a livestock face normalization method that can handle arbitrary face directions. In this study, a lightweight angle detection and region-based convolutional network (LAD-RCNN) was developed, which contains a new rotation angle coding method that can detect the rotation angle and the location of the animal’s face in one stage. LAD-RCNN also includes a series of image enhancement methods to improve its performance. LAD-RCNN has been evaluated on multiple datasets, including a goat dataset and infrared images of goats. Evaluation results show that the average precision of face detection was more than 97%, and the deviations between the detected rotation angle and the ground-truth rotation angle were less than 6.42° on all the test datasets. LAD-RCNN runs very fast and only takes 13.7 ms to process a picture on a single RTX 2080Ti GPU. This shows that LAD-RCNN has an excellent performance in livestock face recognition and direction detection, and therefore it is very suitable for livestock face detection and normalization.

## 1. Introduction

The need for the identification of individual livestock has become an urgent problem with the requirement of quality control, welfare management, and large-scale livestock farming of livestock [1]. Ear tags and radio-frequency identification (RFID) tags are currently commonly used for livestock individual identification, but those tags need to be nailed to the ears or implanted under the skin of livestock, which may reduce the welfare of livestock, and RFID tags can only be read when they are close to RFID reader [2]. In contrast, face recognition technology can realize non-contact livestock identification, which can reduce animal stress. Animal face recognition technology has been widely studied in animal husbandry [3], especially in the field of pig, cattle and sheep, and goat face recognition [1,4,5].

Face recognition consists of three sub-tasks: face detection, face normalizing, and face identification [6,7]. Among them, face detection is to detect the location of a face in an image; face normalizing is to align the faces to normalized coordinates; and face identification is implemented on normalized faces [6]. Most livestock face recognition studies focus on face identification, and there are some studies that focus on face detection, such as Shuang Song [8], who detected sheep faces with Pruning-Based YOLOv3; Billah et al. [4] detected the goat face location with YOLO V4; Hitelman et al. [9] detect the sheep face location through Faster RCNN; and Wang and Liu [10] detected pig face location through EfficientDet-D0. However, no study was found on livestock face normalization, and livestock face recognition technology is still in the research stage and has not yet been applied in livestock farms.

Face recognition without face normalization will significantly reduce performance [11]. The keypoint detection method is widely used in human face normalization [7], and the face image is normalized by affine transformations such as rotation and scaling according to detected keypoints [12]. In the scene of intelligent livestock monitoring, the camera is generally placed above the livestock house, and livestock may not cooperate when taking photos. So, the captured image may contain a face with an arbitrary direction. However, the keypoint detection may fail when the face rotation angle is too large, as tested on dlib’s shape detector [13]. To address this gap in research, a lightweight angle detection and region-based convolutional network (LAD-RCNN) was proposed in this study. LAD-RCNN realizes face location detection and face rotation angle detection in an arbitrary direction in one stage. Livestock face normalization will be conveniently achieved through clipping, rotation, and scaling according to the face location and rotation angle detected by LAD-RCNN.

The main contributions of this paper are as follows:

(1) A lightweight angle detection and region-based convolutional network (LAD-RCNN) was proposed in this study, which can handle arbitrary directions of livestock faces. LAD-RCNN was evaluated in multiple datasets. The average precision was more than 97%, and the average angle difference between the detection angle and the ground-truth angle was within 6.42°.
(2) A rotation angle coding method was proposed in this study, which could deal with the angle discontinuity problem.
(3) A lightweight backbone for LAD-RCNN was proposed in this study, which is faster than the widely used backbone MobileNetV2, ResNet50, and VGG16 with no significant accuracy reduction. The average detection speed of LAD-RCNN reaches 13.7 ms per image tested on a single GeForce RTX 2080 Ti GPU.
(4) To adapt to livestock research, a dual dataset model for LAD-RCNN was designed in this study so that the dataset without angle data can also be used to train LAD-RCNN, which facilitates the use of various datasets. In addition, LAD-RCNN has a lot of built-in data amplification methods to support the use of small datasets.
(5) The code of LAD-RCNN is open source. The code is available at https://github.com/SheepBreedingLab-HZAU/LAD-RCNN/ (accessed on 19 April 2023). Peers of livestock face recognition research can directly employ LAD-RCNN in their study to realize face detection and normalization with little modification.

The rest of this paper is organized as follows. In Section 2, the related work about object detection is briefly described. In Section 3, the components and evaluation metrics of LAD-RCNN are introduced. The experimental results are described in Section 4. Finally, the discussion and conclusions are drawn in Section 5 and Section 6, respectively.

## 2. Related Work

### 2.1. Object Detection

Object detection can be classified into two categories: “one-stage detection” and “two-stage detection”. The one-stage detection has no region proposal stage and detects the location and classification in one stage; the two-stage detection carries out region proposal first and then carries out classification and location detection. Faster R-CNN [14] and Mask RCNN [15] are currently widely used two-stage object detection methods. Faster R-CNN is developed on the basis of Fast R-CNN [16] by replacing time-consuming selective search with a region proposal network (RPN) to improve detection speed. Mask R-CNN [15] adds a branch for predicting an object mask in parallel with the existing bounding box recognition branch in Faster R-CNN to realize the segmentation task and replaces ROI Pooling with ROI Align to improve the performance of the segmentation task. SSD [17] is the first widely used one-stage object detection method, which uses multi-scale feature maps to detect objects of different sizes. RetinaNet [18] introduced focal loss to solve the problem of “imbalance between positive and negative samples” in one-stage object detection, which improves the detection accuracy; YOLO V4 [19] is a commonly used one-stage detection model. YOLO V4 is developed based on previous versions of YOLO models [20,21,22] and introduces a series of features to increase detection accuracy. In recent years, it has also been reported that the transformations, which have been widely used in natural language processing (NLP), have been used for object detection [23,24,25].

These methods have excellent performance in object detection. However, these methods cannot detect the rotation angle, so they cannot be used for the task of livestock face normalization.

### 2.2. Angle-Based Rotated Object Detection

Angle-based rotated object detection methods have developed rapidly in aerial object detection and text detection, and it is developed by adding an angle detection into the object detection and usually represented as a vector (*x*, *y*, *w*, *h*, *θ*) [26]. Since the performance of the two-stage detector is better than the one-stage detector in rotated object detection, most of the rotated object detector relies on the two-stage RCNN frameworks by replacing anchors and RoI pooling with rotation anchors and rotated RoI pooling [27,28,29]. The characters of aerial images are small and densely packed, which is hard to detect [30]. In order to have better performance in aerial object detection, R^2^PN [31] generates anchors in multiple directions by controlling scale, rations, and angle and redefines the IoU computation method. SCRNet [32] proposes a multi-dimensional attention network to reduce noise interference and improve the sensitivity to small objects and adds an IoU constant factor to the loss function so that the loss function can better handle rotating bounding box regression. Yang and Yan [33] deal with angle prediction questions using classification to alleviate the discontinuous boundary problem and propose circular smooth label (CSL) technology to detect large aspect ratio objects. ReDet [34] proposes a rotation-equivariant backbone to extract rotation-equivariant features and a rotation-invariant RoI Align to obtain rotation-invariant features, which reduces the number of parameters. Oriented R-CNN [35] proposes an oriented region proposal network (oriented RPN) that generates oriented proposals on fewer anchors, which improves the detection speed. MRDet [29] proposes an arbitrary-oriented region proposal network (AO-RPN) that adds a branch in PRN to learn transformation parameters for generating oriented proposals; SCRDet++ [36] extends SCRDet through instance-level denoising modules to improve the performance of small and densely packed object detection.

**Figure 1:**
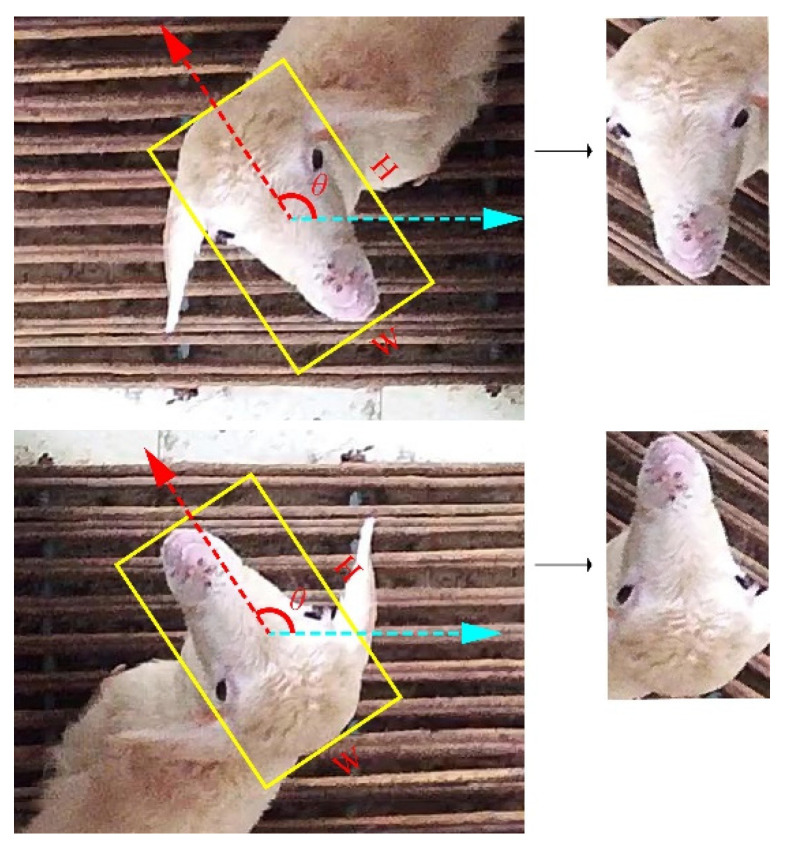
Angle encoding method used in another study [26]. The angle is represented as the angle between the long axis and the horizontal axis. In this way, an inverted face image may be obtained. Therefore, the angle encoding method in the other study is not suitable for animal face recognition and normalization.

These angle-based rotated object detectors have excellent performance in aerial object detection and text detection. However, the rotation angle is represented as the angle between the long axis and the horizontal axis in other studies [26], which may obtain a reversed result in normalizing livestock faces (Figure 1). Therefore, it is necessary to design new rotation angle representation methods and then propose a new angle detection and region-based convolutional network suitable for livestock face normalization.

## 3. Method

### 3.1. Model

This study sets a series of preset boxes (anchors) with specific sizes and positions distributed in each region of the image. Each anchor corresponds to nine values in the feature map that were extracted from the image. After supervised learning, anchors corresponding to the object can be picked out, and the rotation angle and location of the object can be calculated through the head network. Face detection and normalization can be realized through rotation and cropping according to the detected position and rotation angle.

#### 3.1.1. Anchors

A series of anchors was set associated with cells in feature maps inspired by [17]. The center point of the anchor is determined by the position of the associated cell. The initial size of the anchor is preset, and the anchor associated with the shallow feature is smaller than the deep feature. Each cell in feature maps is associated with *k* (*k* = 6 in default) anchors. Six anchors per cell were set, controlled by two scaling ratios $\left(1,\;\sqrt{2}\right)$ and three aspect ratios $\left(1.0,\;2.0,\;0.5\right)$ by default. The center of the anchors coincides with the center of the associated cell. The anchor is used to encode or decode box location, which is described in Section 3.1.6. The total number of anchors is related to the pixels of the input image. When the pixels of the input image are 400 × 400, 20,058 anchors will be generated.

#### 3.1.2. Overall Structure

**Figure 2:**
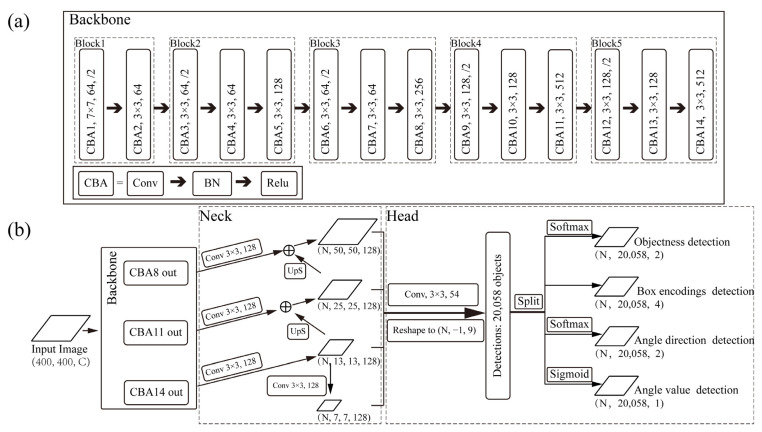
The overall pipeline of the LAD-RCNN: (**a**) backbone network; (**b**) overall pipeline of the LAD-RCNN. The rounded rectangle represents the operation on the tensor; the rhombus represents the tensor; CBA represents the sequential operation of convolution, normalization, and ReLU activation; ⊕ represents the add operation; and UpS indicates the up-sampling operation.

LAD-RCNN is designed inspired by SSD [17] and Faster RCNN [14]. The overall structure of LAD-RCNN is depicted in Figure 2. Three tensors are generated from the input image by the backbone network. After convolution, up-sampling, and addition operations, four feature maps with different sizes are generated from those three tensors. The 4 feature maps are convoluted with the same kernel to output tensors with 54 channels (each cell corresponds to 6 anchors, and each anchor corresponds to 9 numbers). Output tensors generated from four feature maps are concatenated and reshaped to a tensor with nine channels. Among them, two channels are used for objectness detection, four channels are used for box encodings detection, two channels are used for angle direction detection, and one channel is used for angle value detection.

#### 3.1.3. Backbone

The backbone network is used to extract information from input images for the neck network-generating feature maps. The backbone network of LAD-RCNN consists of 14 sequential CBA, and each CBA consists of a convolution layer, a batch normalization layer, and an activation layer (Figure 2). The first CBA uses 7 × 7 kernels in convolution, and the other CBA uses 3 × 3 kernels. The backbone network is divided into five blocks. In each block, the step length is two in the first convolution layer. In the last four blocks, the dimension of the output tensor of the third CBA is four times the size of the first two CBA. The output tensor of the last CBA in the last three blocks is transferred to the neck network.

#### 3.1.4. Rotation Angle

**Figure 3:**
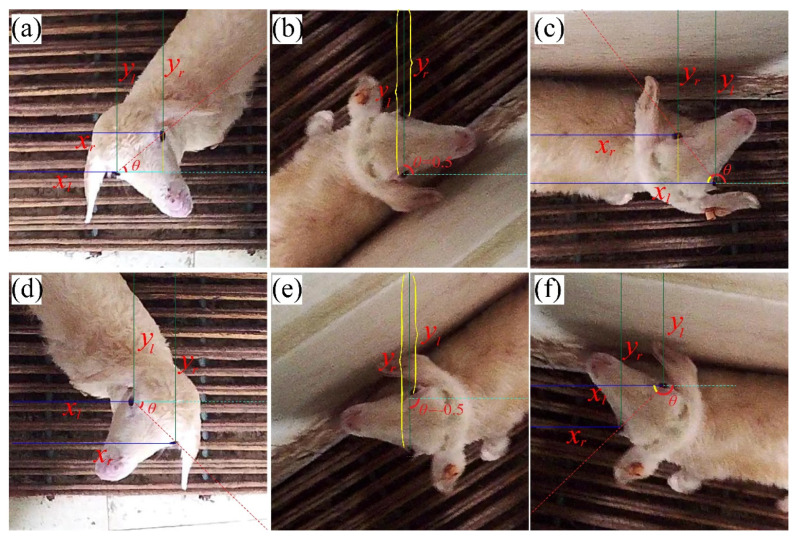
Illustration of angle definition. The left eye in the normalized picture was chosen as the left keypoint, and the right eye was chosen as the right keypoint; (**a**,**d**) correspond to the condition ($x_{r}-x_{l}>0)$, where *θ* is between [0, 0.5) in (**a**) and between (0, −0.5) in (**d**); (**b**) corresponds to the condition $\left(y_{l}-y_{r}>0,x_{r}-x_{l}=0\right)$, where *θ* = 0.5; (**c**) corresponds to the condition $\left(y_{l}-y_{r}\geq 0,x_{r}-x_{l}<0\right)$, where *θ* is between (0.5, 1]; (**e**) corresponds to the condition $\left(y_{l}-y_{r}<0,x_{r}-x_{l}=0\right)$, where *θ* = −0.5; (**f**) corresponds to the condition $\left(y_{l}-y_{r}<0,x_{r}-x_{l}<0\right)$, where *θ* is between (−0.5, −1).

The angle between the horizontal axis and the line from the left keypoint to the right keypoint was used to represent the rotation angle of the object. These two keypoints can be selected empirically by the principle that the line from the left keypoint to the right should be parallel to the horizontal axis in the standardized object. The floating number between (−1.0, 1.0] was used to represent the rotation angle between (−180°, 180°], where a positive value indicates counterclockwise rotation and a negative value indicates clockwise rotation. The calculation method for the rotation angle (*θ*) is shown in Formula (1), Formula (2), and Figure 3.

(1) $$k=\left\{\begin{array}{c} \frac{y_{l}-y_{r}}{x_{r}-x_{l}}\;\;\;\;\;\;\;\;\;\;\;\;\;\;\;\;\;\;\;\;\;\;\;\;\;\;\left(x_{r}\neq x_{l}\right)\\ \left(y_{l}-y_{r}\right)\times \infty \;\;\;\;\;\;\;\;\;\;\;\;\;\;\left(x_{r}=x_{l}\right) \end{array}\right.$$

(2) $$\theta =\left\{\begin{array}{l} \frac{\tan^{-1}\left(k\right)}{\pi }\;\;\;\;\;\;\;\;\;\;\;\;\;\;\;\;\;\;\;\;\;\;\;\;\;\left(x_{r}-x_{l}>0\right)\\ 0.5\;\;\;\;\;\;\;\;\;\;\;\;\;\;\;\;\;\;\;\;\;\;\;\;\;\;\;\;\;\;\;\;\left(y_{l}-y_{r}>0,x_{r}-x_{l}=0\right)\\ 1-\frac{\tan^{-1}\left(\left|k\right|\right)}{\pi }\;\;\;\;\;\;\;\;\;\;\;\;\;\;\;\;\left(y_{l}-y_{r}\geq 0,x_{r}-x_{l}<0\right)\\ -0.5\;\;\;\;\;\;\;\;\;\;\;\;\;\;\;\;\;\;\;\;\;\;\;\;\;\;\;\;\;\left(y_{l}-y_{r}<0,x_{r}-x_{l}=0\right)\\ \frac{\tan^{-1}\left(\left|k\right|\right)}{\pi }-1\;\;\;\;\;\;\;\;\;\;\;\;\;\;\;\;\left(y_{l}-y_{r}<0,x_{r}-x_{l}<0\right) \end{array}\right.$$

Here, $x_{l}$ is the distance between the left keypoint and the left frame of the picture; $x_{r}$ is the distance between the right keypoint and the left frame of the picture; $y_{l}$ is the distance between the left key point and the upper frame of the picture; $y_{r}$ is the distance between the right key point and the top frame of the picture.

#### 3.1.5. Angle Discontinuity Problem

**Figure 4:**
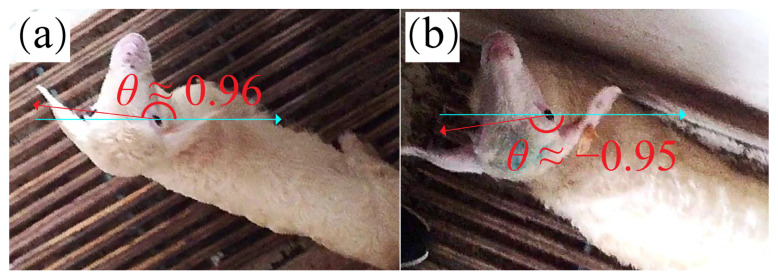
Angle discontinuity problem. The difference in rotation angles in (**a**,**b**) is little, but the difference in calculated *θ* is very large.

The difference in rotation angle is little between the object rotating counterclockwise by nearly 180° $\left(\theta \to 1.0\right)\;$ and the object rotating clockwise by nearly 180°$\;\left(\theta \to -1.0\right)$, but the difference in the calculated *θ* is very large (Figure 4). It may cause the model not to converge in training. To deal with this problem, the angle value *θ* was split into its absolute value and its sign based on the reason that its absolute value is continuous. Therefore, LAD-RCNN detects the absolute angle value and the direction of rotation, respectively.

#### 3.1.6. Head Network

Each anchor box corresponds to nine values (Figure 2), in which four values are used to detect the box, two values are used to detect objectness, two values are used to detect rotation direction, and one value is used to detect absolute angle value.

The box location was decoded through bounding box regression [14,37]:

(3) $$\begin{array}{c} \begin{array}{ll} x=\left(t_{x}/10.0\right)\times w_{a}+x_{a} & y=\left(t_{y}/10.0\right)\times h_{a}+y_{a}\\ w=e^{\left(t_{w}∕5.0\right)}\times w_{a} & h=e^{\left(t_{h}∕5.0\right)}\times h_{a} \end{array} \end{array}$$

where *x*, *y*, *w*, and *h* denote the predicted box’s center coordinates and its width and height, respectively; *t_x_*, *t_y_*, *t_w_*, and *t_h_* denote the output tensors of the CNN; *x_a_*, *y_a_*, *w_a_*, and *h_a_* denote the anchor’s center coordinates and its width and height, respectively.

Accordingly, the ground-truth box was encoded as follows:

(4) $$\begin{array}{c} \begin{array}{ll} t_{x}^{*}=10.0\times \frac{\left(x^{*}-x_{a}\right)}{w_{a}}, & t_{y}^{*}=10.0\times \frac{\left(y^{*}-y_{a}\right)}{h_{a}},\\ t_{w}^{*}=5.0\times log\left(\frac{w^{*}}{w_{a}}\right), & t_{h}^{*}=5.0\times log\left(\frac{h^{*}}{h_{a}}\right) \end{array} \end{array}$$

where x*, y*, w*, and h* denote the ground-truth box’s center coordinates and its width and height, respectively.

The objectness detection and rotation direction detection results were converted through the SoftMax function:

(5) $$Softmax\left(z_{j}\right)=\frac{e^{z_{j}}}{\sum_{i}e^{z_{i}}}$$

where $i\;\mathrm{and}\;j\in \left\{0,\;1\right\}$, and $z_{j}$ denotes the *j*-th value. $Softmax\left(z_{j}\right)$ denotes the calculated probability.

The rotation angle value was calculated through a sigmoid function:

(6) $$Sigmoid\left(x\right)=\frac{1}{1+e^{-x}}$$

### 3.2. Training

#### 3.2.1. Dual Dataset Training

To facilitate the use of various datasets to train LAD-RCNN, LAD-RCNN is designed to be trained by datasets both with angle data (Dataset 1) and without angle data (Dataset 2). Dataset 1 contains at least the list of x-axis minimum values, x-axis maximum values, y-axis minimum values, y-axis maximum values, and rotation angle value of all labeled boxes; Dataset 2 contains at least the list of x-axis minimum values, x-axis maximum values, y-axis minimum values and y-axis maximum values of all labeled boxes.

**Figure 5:**
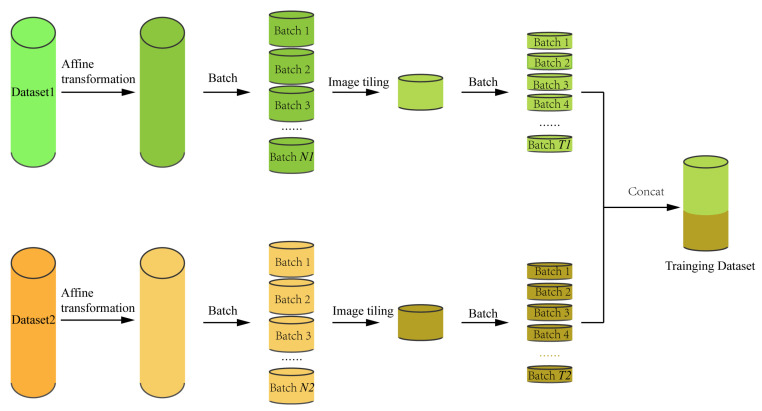
The generation pipeline of training dataset in LAD-RCNN. Affine transformation represents counterclockwise rotation by 90°, horizontally flipping or vertically flipping with a preset probability; N1 and N2 are determined by the preset probability of image merge in Dataset 1 and Dataset 2, respectively; image tiling represents the operation that generates one image from each batch to form a new dataset. T1 and T2 are preset batchsizes of Dataset 1 and Dataset 2, respectively.

Dataset 1 is mainly used to train the rotation angle and rotation direction; Dataset 2 is mainly used for objectness detection and box encodings detection. It should be noted that if all data contains angle information, Dataset 2 can be the same as Dataset 1. The generation pipeline of the training dataset is depicted in Figure 5.

#### 3.2.2. Loss Function

The overall loss function of LAD-RCNN is the weighted sum of object localization loss, objectness loss, absolute angle value loss, and angle direction loss:

(7) $$L_{LAD-RCNN}=\lambda_{loc}L_{loc}+\lambda_{obj}L_{obj}+\lambda_{av}L_{av}+\lambda_{ad}L_{ad}$$

where *λ_loc_*, *λ_obj_*, *λ_av_,* and *λ_ad_* are the trade-off parameters and are set to 1.0, 5.0, 1.0, and 10.0 by default, respectively. *L_loc_* denotes localization loss; *L_obj_* denotes objectness loss; *L_av_* denotes absolute angle value loss; *L_ad_* denotes angle direction loss.

Mini-batch sampling [16] was employed to deal with the imbalance between positive and negative samples in training. Localization loss is defined as follows:

(8) $$L_{loc}=\frac{1}{N_{loc}}\sum_{i=1}^{N_{loc}}\sum_{j\in \left\{x,y,w,h\right\}}\mathrm{Huber}\left(t_{i,j}^{*}-t_{i,j}\right)$$

In which,

(9) $$\mathrm{Huber}\left(a\right)=\left\{\begin{array}{cc} 0.5a^{2} & \left(\left|a\right|\leq \delta \right)\\ \delta \left|a\right|-0.5\delta^{2} & \left(\left|a\right|>\delta \right) \end{array}\right.$$

Here, $N_{loc}$ is the number of positive anchors in a mini-batch; *i* is the index of positive anchors in a mini-batch; *x*, *y*, *w*, and *h* are the same as in Formula (1). $t_{i,j}^{*}$ is the ground-truth value of *j*, corresponding to the *i*-th anchor calculated by Formula (1); $t_{i,j}$ is the predicted value of *j*, corresponding to the *i*-th anchor calculated by Formula (1); *δ* is a variable in the Huber function, and we set *δ* = 1 by default.

Objectness loss is defined as follows:

(10) $$L_{obj}=\frac{1}{N_{obj}}\sum_{i=1}^{N_{obj}}\mathrm{FL}\left(1-\left|p_{i}-p_{i}^{*}\right|\right)$$

In which,

(11) $$\mathrm{FL}\left(p_{t}\right)=-{\left(1-p_{t}\right)}^{\gamma }\log\left(p_{t}\right)$$

Here, $N_{obj}$ is the number of anchors in a mini-batch, *i* is the index of anchor in a mini-batch, and $p_{i}$ is the predicted probability that the *i*-th anchor is marked as an object. $p_{i}^{*}$ indicates whether the *i*-th anchor box is marked as an object. When the *i*-th anchor is marked as an object, $p_{i}^{*}=1$; otherwise, $p_{i}^{*}=0$. FL(*) is focal loss function [18], and we set γ = 2 by default.

Absolute angle value loss is defined as follows:

(12) $$L_{av}=\frac{1}{N_{loc}}\left(\sum_{i=1}^{N_{loc}^{ds1}}\lambda_{ds1}\times 0.5{\left(\theta_{v,i}^{*}-\theta_{v,i}\right)}^{2}+\sum_{i=1}^{N_{loc}^{ds2}}\lambda_{ds2}\times 0.5{\left(\theta_{v,i}\right)}^{2}\right)$$

Here, $N_{loc}$ is the number of positive anchors in a mini-batch, *i* is the index of positive anchors in a mini-batch, and $N_{loc}^{ds1}$ is the number of positive anchors corresponding to Dataset 1, which is with angle data. $N_{loc}^{ds2}$ is the number of positive anchors corresponding to Dataset 2, which is without angle data. $\theta_{v,i}$ is the predicted absolute angle value of the *i*-th anchor; $\theta_{v,i}^{*}$ is the ground-truth absolute angle value of the *i*-th anchor; $\lambda_{ds1}$ and $\lambda_{ds2}$ are the trade-off parameters and are set to 10.0 and 0.0 by default, respectively.

Angle direction loss is defined as follows:

(13) $$L_{ad}=\frac{1}{N_{ad}}\underset{i\in I}{\sum }\mathrm{FL}\left(1-\left|p_{\theta ,i}-p_{\theta ,i}^{*}\right|\right)$$

In which,

(14) $$I=\left\{i|i\in A,\left|\theta_{v,i}^{*}\right|>\varepsilon \right\}$$

Here, $N_{ad}$ is the number of elements in set I. FL(*) is the focal loss function defined by Formula (11); $p_{\theta ,i}$ is the predicted probability that the *i*-th anchor has a counterclockwise rotation; $p_{\theta ,i}^{*}$ indicating the ground-truth probability of whether the *i*-th anchor box has a counterclockwise rotation. When the *i*-th anchor is marked as having a counterclockwise rotation, $p_{\theta ,i}^{*}$ = 1; otherwise, $p_{\theta ,i}^{*}$ = 0. *A* is the set of all anchors; $\theta_{v,i}^{*}$ is the ground-truth absolute angle value of the *i*-th anchor; *ε* is a preset parameter with default value of 0.025.

#### 3.2.3. Data Augmentation

To make LAD-RCNN more robust to arbitrary rotation angles and suitable for small datasets, the training set can be randomly operated by the following operations:

**Counterclockwise rotation by 90°**. The ground-truth angle after rotation can be calculated as follows:

(15) $$\theta^{\prime }=\left\{\begin{array}{c} \frac{\theta \times 180+90}{180}\;\;\;\;\;\;\;\;\left(\theta \leq 0.5\right)\\ \frac{\theta \times 180-270}{180}\;\;\;\;\;\;\left(\theta >0.5\right) \end{array}\right.$$

where *θ* is the original angle, and *θ′* is the angle after the operation; the same applies below.

**Horizontally flipping**. The ground-truth angle after horizontally flipping can be calculated as follows:

(16) $$\theta^{\prime }=-\theta$$

**Vertically flipping**. The ground-truth angle after vertically flipping can be calculated as follows:

(17) $$\theta^{\prime }=\left\{\begin{array}{l} \left|\theta \right|-1\;\;\;\;\;\;\left(\theta <0\right)\\ 1-\theta \;\;\;\;\;\;\;\;\left(\theta \geq 0\right) \end{array}\right.$$

**Image tiling**. During the training, the images are tiled together with a preset probability. The images are tiled by combining 4 images into 1 image in the form of 2 × 2 or combining 9 images into 1 image in the form of 3 × 3.

All data augmentation methods can be conveniently achieved by adjusting the parameters in a config file. These operations are independent of each other, and trigger probability can be set separately for each operation. Thus, an image may be operated in multiple ways in training. The set parameters for training by evaluating datasets are described in Section 4.2 and Section 4.3.

### 3.3. Evaluation Metrics

The performance of LAD-RCNN was measured by precision, recall rate, F1-score, average precision, and average angle difference (AAD).

(18) $$Precision=\frac{TP}{TP+FP}$$

(19) $$Recall=\frac{TP}{TP+FN}$$

(20) $$F_{1}-\mathrm{score}=2\times \frac{Precision\times Recall}{Precision+Recall}$$

where *TP*, *FP*, and *FN* are the number of true positive, false positive, and false negative prediction boxes at IoU = 0.5, respectively.

AP is the area under the precision–recall curve, which is widely used in object detection evaluation [38]. The calculating formula is as follows:

(21) $$AP=\int_{0}^{1}p\left(r\right)dr$$

where *r* represents the recall rate, and *p*(*r*) is the precision when the recall rate is *r*.

The performance of angle detection of LAD-RCNN was measured by the average angle difference (*AAD*) between the detection angle and the ground-truth angle:

(22) $$AAD=\frac{1}{N_{obj}}\left(\sum_{i=1}^{N_{obj}}D\left(\theta_{i}^{*},\;\theta_{i}\right)\right)\times 180{}^{\circ}$$

In which,

(23) $$D\left(\theta_{i}^{*},\;\theta_{i}\right)=\left\{\begin{array}{c} \left|\theta_{i}^{*}-\theta_{i}\right|\;\;\;\;\;\;\;\;\;\;\;\;\;\;\;\left(\left|\theta_{i}^{*}-\theta_{i}\right|<1\right)\\ 2-\left|\theta_{i}^{*}-\theta_{i}\right|\;\;\;\;\;\;\;\;\left(\left|\theta_{i}^{*}-\theta_{i}\right|\geq 1\right) \end{array}\;\;\right.$$

where $N_{obj}$ is the total number of objects detected in the test set; θ_i_* is the ground-truth angle with direction corresponding to the *i*-th detected objects; *θ_i_* is the predicted angle with direction in the *i*-th detected objects.

## 4. Evaluation Result

### 4.1. Backbone Evaluation

The backbone architecture of LAD-RCNN is shown in Figure 2. In addition to our backbone, LAD-RCNN also supports the use of other backbone networks, such as MobileNetV2, VGG16, ResNet50, etc. MobileNetV2 [39] is a lightweight network designed for mobile users; VGG16 is a classic backbone network; and ResNet50 is a widely used deep convolutional network. Compared to ResNet50 and MobileNetV2, our backbone has fewer layers; compared to VGG16, our network has fewer channels per layer. The first layer of our backbone uses the large kernel of 7 × 7 to increase the receptive field; the other layers use small kernels of 3 × 3 to reduce model size. These designs may improve detection speed.

Table 1 shows the comparison between our backbone, MobileNetV2, VGG16, and ResNet50. The results show that the number of parameters in our backbone is far less than that of VGG16 and ResNet50, which is like the lightweight network MobileNetV2, and the detection speed of LAD-RCNN with our backbone was 72.74FPS (13.7 ms per image), which was 36.29%, 32.15%, and 64.12% faster than that of LAD-RCNN with MobileNetV2, VGG16, and ResNet50, respectively.

### 4.2. Experiments on Goat Dataset

The goat dataset [4] labeled the location of the goat face and eyes, which contains 1680 training data and 1311 test data. There are 438 and 613 images containing two eyes in the training set and test sets, respectively. The face rotation angle was calculated according to the location of the two eyes. Dataset 1, which contains angle information, was generated by training data containing rotation angle. Dataset 2, without angle information, was generated by all the training data.

The probabilities of data augmentation operations on the goat dataset were set as outlined in Table 2. The batchsize of Dataset 1 with angle data was set to 7, and that of Dataset 2 without angle data was set to 5. The input image channel was set to three. The total training step was set to 50,000.

**Figure 6:**
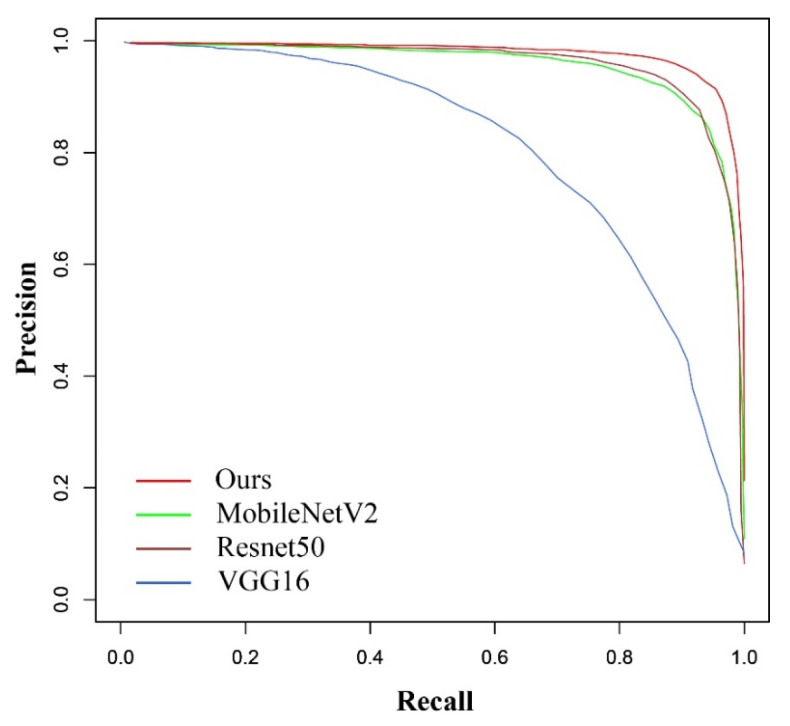
Precision–recall curves on goat dataset.

**Figure 7:**
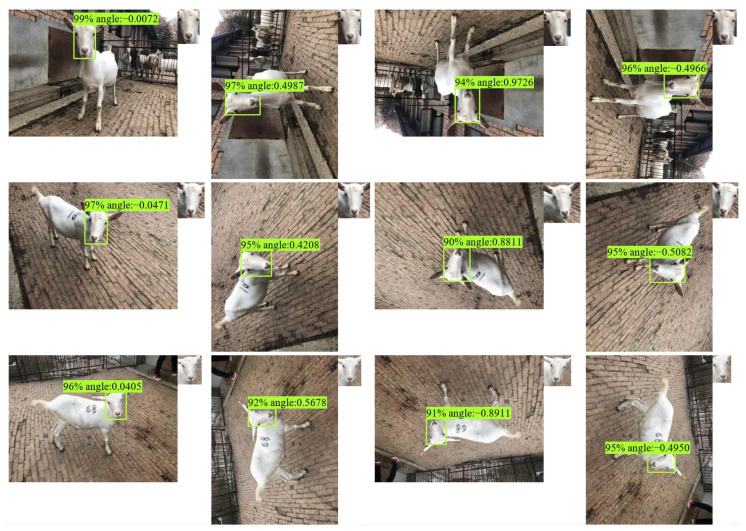
Detection examples on goat image with LAD-RCNN. The small image in the upper right corner of each image is the extracted normalized face according to the detection result. The four pictures in each line represent the same picture in the test set, which are the original image and images rotated by 90°, 180°, and 270°, respectively.

The 613 images in test data containing angle information were used to evaluate the trained model. To evaluate the performance of LAD-RCNN on detecting goat face with arbitrary rotation angles, the test image was rotated by 90°, 180°, and 270°, respectively, to form a new test dataset with 613 × 4 images. The test results (Table 3, Figure 6 and Figure 7) show that the AP values were more than 97% when ours, MobileNetV2, or ResNet50 were adopted as the backbone network, and the AP was the highest when our backbone was adopted. When ours, MobileNetV2, or ResNet50 were used as the backbone network, the average angle difference was within 6.42°.

**Figure 8:**
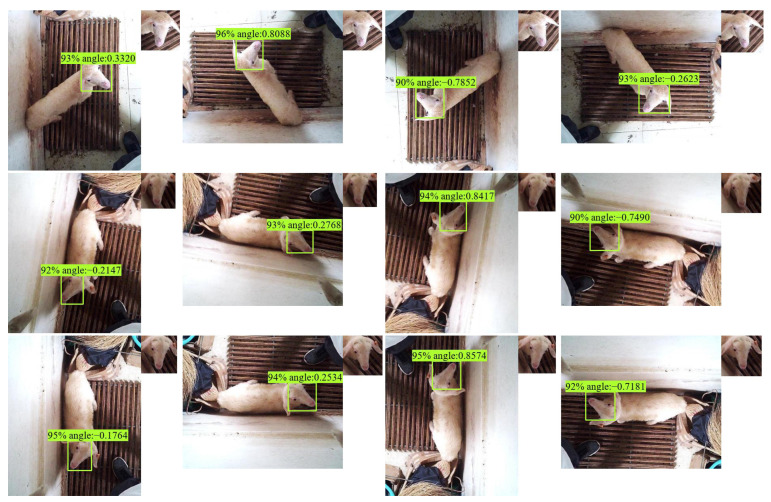
Detection examples on sheep bird-view image with LAD-RCNN. The small image in the upper right corner of each image is the extracted normalized face according to the detection result. The four pictures in each line represent the same picture in the test set, which are the original image and images rotated by 90°, 180°, and 270°, respectively.

The model trained by the goat dataset also performs well in detecting and normalizing faces in sheep bird’s-eye view images (Figure 8).

### 4.3. Experiments on Goat Infrared Image Dataset

The self-made goat infrared image dataset labeled the location of the goat face and the rotation angle of the goat face, which contains 2409 training data and 1000 test data. Dataset 1, containing angle information, and Dataset 2, without angle information, were both generated from all the training data.

The probabilities of data augmentation operations on the goat infrared image dataset were set as outlined in Table 4. The batchsize of Dataset 1 was set to 7, and that of Dataset 2 was set to 5. The input image channel was set to one. The total training step was set to 50,000.

**Figure 9:**
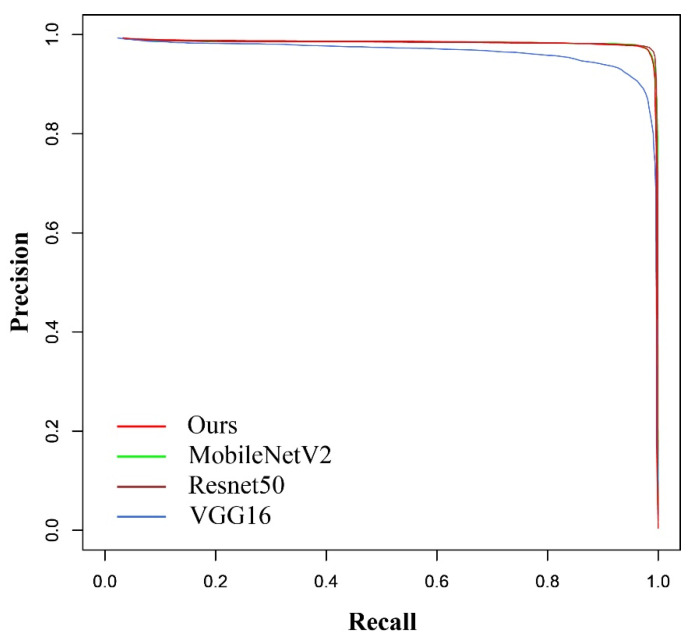
Precision–recall curves on goat infrared image dataset.

**Figure 10:**
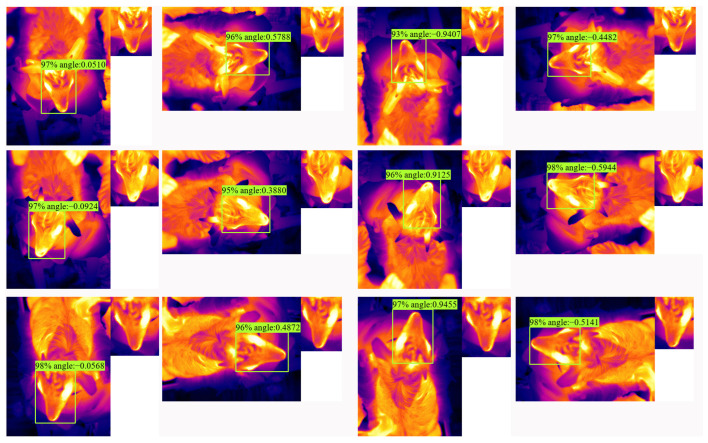
Detection examples on goat infrared image with LAD-RCNN. The small image in the upper right corner of each image is the extracted normalized face according to the detection result. The four pictures in each line represent the same picture in the test set, which are the original image and images rotated by 90°, 180°, and 270°, respectively.

To evaluate the performance of LAD-RCNN on detecting goat face with arbitrary direction in an infrared image, the test image was rotated by 90°, 180°, and 270°, respectively, to form a new test dataset with 4000 images. The test results (Table 5, Figure 9 and Figure 10) show that all the AP were more than 96%, and all the average angle differences were within 5.94°. When ours, MobileNetV2, or ResNet50 were adopted as the backbone network, the AP values were more than 98%, and the average angle differences were within 4.96°.

## 5. Discussion

Livestock face recognition can realize non-contact livestock identification and improve animal welfare. With the demand for standardized large-scale livestock farming, a lot of research in the area of livestock face recognition was conducted on pigs, cattle, sheep, and other livestock [1,3,4,5]. Face recognition consists of three sub-tasks: face detection, face normalizing, and face identification [6,7]. Most livestock face recognition studies focus on face identification, and there are some studies that focus on face detection. However, no study was found on livestock face normalization. Face recognition without face normalization will significantly reduce performance [11]. To address this gap in research, a new approach has been developed called the lightweight angle detection and region-based convolutional network (LAD-RCNN) for livestock face detection and normalization. LAD-RCNN is capable of detecting livestock faces and their rotation angles with arbitrary directions in one stage, making it a highly efficient tool for researchers.

In the scenes of livestock automatic monitoring, real-time monitoring of livestock is required. Therefore, face detection and normalization should be completed as soon as possible. Compared with the two-stage method, the one-stage object detector gets rid of the time-consuming regional proposal step and directly detects objects from the densely predesigned candidate boxes, which has faster detection speed [29]. Lin et al. [18] propose focal loss to solve the problem of “imbalance between positive and negative samples” in a one-stage object detector and so that the one-stage detector can achieve good performance in rotated object detection [40]. In addition, due to the poor performance of face recognition through too small face images, it is low value to detect too small objects in the field of livestock face recognition. That is, it is only needed to detect the face and its direction with normal size in the field of livestock face recognition. Therefore, LAD-RCNN was designed with a one-stage strategy.

A lightweight backbone for LAD-RCNN was designed in this study. The evaluation results on multiple datasets show that when using LAD-RCNN with our backbone to detect faces with arbitrary directions, the AP was more than 97%, and the average angle differences between the detection angle and the ground-truth angle were within 6.42° (Table 3 and Table 5). The backbone evaluation results show that the number of parameters in our backbone is 5.21 times and 8.36 times less than that in VGG16 and ResNet50, respectively, and the detection speed of our backbone is 47%, 104%, and 150% faster than MobileNetV2, VGG16, and ResNet50, respectively. Therefore, the backbone proposed in this study improves the detection speed without reducing the detection accuracy.

Infrared thermal imaging technology is a fast non-contact temperature measurement technology that can generate images based on surface temperature information and provide dynamic information of surface temperature changes caused by physiological processes. It has been widely used in animal research [41,42,43,44,45]. Based on the characteristics of infrared images, it was speculated that animal recognition in infrared images would become one of the research hotspots. In order to adapt LAD-RCNN to infrared images with a single channel, a channel number configuration interface was added to the config file of LAD-RCNN. LAD-RCNN will adapt to infrared thermal images if the channel number is set to one. The test results on goat infrared image (Table 5, Figure 9 and Figure 10) shows that LAD-RCNN performs well in face detection on infrared images.

In the field of animal research, a small dataset may be required to be used for face recognition for some reasons [46,47]. In order to perform better in small datasets, LAD-RCNN integrates some dataset enhancement functions, such as horizontal flip, vertical flip, 90° rotation, 2 × 2 merger, and 3 × 3 merger. Horizontal flipping, vertical flipping, and 90° rotation can make the livestock face directions in the training dataset more diverse, and the closer the probability of operation is to 0.5, the greater the diversity. The merge operation refers to concatenating multiple images into one image. The higher the probability, the higher the diversity of the dataset. The training set of the goat dataset only contains 1680 data, of which only 438 data contain rotation angle information. The evaluation results for this dataset show that the AP reached 97.55%, and the average angle difference between the detection angle and the ground-truth angle was within 6.42°, which proves that LAD-RCNN performs well in the small dataset.

It is a pity that no more livestock datasets have been found for extensive verification of LAD-RCNN due to most of the livestock recognition studies have not published their labeled dataset. The evaluation result in multiple datasets proves the extensive applicability of LAD-RCNN in various datasets. The experimental conditions tested on all the datasets have been reported in detail in this paper. Peers of livestock face recognition researchers may accelerate their research by directly employing LAD-RCNN in their study to realize face detection and normalization. With the acceleration of livestock face recognition research, face recognition technology will be applied in livestock farms more quickly to get rid of the hurt to livestock caused by ear tags and improve animal welfare.

LAD-RCNN actually provides a tool that can synchronously detect object position and rotation angle. In theory, it can be employed by any study which needs to synchronously detect object position and rotation angle, such as text detection.

## 6. Conclusions

A lightweight angle detection and region-based convolutional network (LAD-RCNN) was proposed in this study for livestock face detection and normalization, which can detect the livestock face and rotation angle with arbitrary directions in one stage. The backbone proposed by this study is a lightweight network, and the detection speed of our backbone is 13.7 ms per image, which is faster than that of MobileNetV2, VGG16, and ResNet50. LAD-RCNN has been evaluated on multiple datasets, and the AP was more than 97%, while the average angle difference between the detection angle and the ground-truth angle was within 6.42°. One of the notable features of LAD-RCNN is its ability to perform well on small datasets and infrared images with a single channel. This shows that the LAD-RCNN has an excellent performance in livestock face detection and angle-based normalization. Overall, this research shows promise for improving livestock face recognition technology.

## Figures and Tables

**Table 1:** Comparison of backbones.

Backbone	Input Resolution	Parameters	FPS
Ours	400 × 400	2.82 M	72.74
MobileNetV2	400 × 400	2.26 M	53.37
VGG16	400 × 400	14.71 M	55.04
ResNet50	400 × 400	23.59 M	44.32

Note: FPS is the test result, including all steps of LAD-RCNN on a single RTX 2080Ti GPU.

**Table 2:** The probabilities of data augmentation operations on goat dataset.

Data Augmentation Operation	Probabilities in Dataset 1	Probabilities in Dataset 2
Counterclockwise rotation by 90°	0.5	0
Horizontally flipping	0.5	0.5
Vertically flipping	0.5	0.5
Image tiling 2 × 2	0.8	0.8

Note: 0.5 indicates a possibility of 50%; others are similar.

**Table 3:** Test result of LAD-RCNN on goat dataset.

Backbone	Precision	Recall	F1 Score	AP	AAD
Ours	95.02%	90.70%	92.81%	97.55%	6.42°
MobileNetV2	89.23%	90.30%	89.76%	95.25%	4.98°
VGG16	64.89%	79.67%	71.52%	79.80%	9.08°
ResNet50	88.99%	91.64%	90.30%	95.62%	6.12°

Note: AAD represents the average angle difference between the detection angle and the ground-truth angle.

**Table 4:** The probabilities of data augmentation operations on goat infrared image dataset.

Data Augmentation Operation	Probabilities in Dataset 1	Probabilities in Dataset 2
Counterclockwise rotation by 90°	0.5	0
Horizontally flipping	0.5	0.5
Vertically flipping	0.55	0
Image tiling 2 × 2	0.8	0.8

Note: 0.5 indicates a possibility of 50%; others are similar.

**Table 5:** Test result of LAD-RCNN on goat infrared image dataset.

Backbone	Precision	Recall	F1 Score	AP	AAD
Ours	96.43%	98.39%	97.40%	98.19%	4.62°
MobileNetV2	97.20%	97.66%	97.43%	98.35%	4.96°
VGG16	89.95%	96.69%	93.20%	96.30%	5.94°
ResNet50	96.93%	98.83%	97.87%	98.29%	4.48°

Note: AAD represents the average angle difference between the detection angle and the ground-truth angle.

## Data Availability

The code of LAD-RCNN is available at https://github.com/SheepBreedingLab-HZAU/LAD-RCNN/ (accessed on 19 April 2023).
